# Perception Space—The Final Frontier

**DOI:** 10.1371/journal.pbio.0030137

**Published:** 2005-04-12

**Authors:** Lars Chittka, Axel Brockmann

## Abstract

Mapping the complex sensory behavior of animals, such as smell in bees, to relevant neural activity provides clues into how animals perceive and respond to the world through their senses

Jakob von Uexküll coined the term *Umwelt* to describe the subjective world of animals. The world that animals perceive is not an objective, veridical representation of the physical world, he argued, but is instead a product of the particular sense organs that each species has acquired in its evolutionary history [1]. Many animals have sensory abilities that humans don't, such as a magnetic compass sense in birds [2] or sensitivity to electric fields in fish [3]. But even within sensory modalities shared by many animals, such as vision, hearing, and olfaction, there are strong differences between species. For example, bees, but not humans, can see UV light [4,5] and smell carbon dioxide [6], and bats can hear ultrasound [7]. But what exactly is the structure of the perceptual worlds proposed by von Uexküll? Can we draw them on paper in the form of maps, allowing us to visualize a particular animal's subjective view of the world? Will such maps allow us to predict the similarity of two stimuli (e.g., two colors or two scents) by inspecting the distance between the loci they produce in a perceptual space? Does understanding the metrics of such maps help us predict how stimulus mixtures will be perceived?

Even though perceptual sensations may strike us as ethereal, they must be based on patterns of activity in neuronal hardware—thus, we need to look into the brains of animals to see how the neuronal circuitry processes the information from the sense organs. The study by Guerrieri et al. [8] on odor space in honeybees in this issue is an excellent example of how behavioral studies can be paired with neurobiological data to access the perceptual space of an animal. Here, we contrast the complexity of scent perception with two examples of simpler perceptual worlds, vertebrate frequency perception and bee color vision.

## The Perception of Pitch in the Auditory System

**Figure 1:**
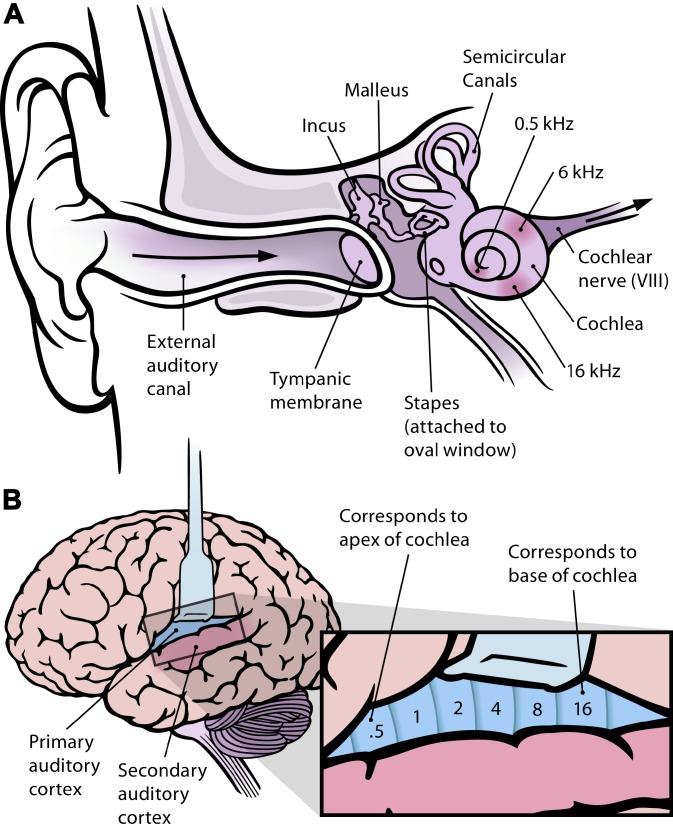
Frequency Coding in the Human Ear and Cortex (A) The human ear and frequency mapping in the cochlea. The three ossicles incus, malleus, and stapes transmit airborne vibration from the tympanic membrane to the oval window at the base of the cochlea. Because of the mechanical properties of the basilar membrane within the snail-shaped cochlea, high frequencies will produce a vibration peak near the oval window, whereas low frequencies will stimulate receptors near the apex of the cochlea (locations for three frequencies indicated schematically). Information from the cochlear receptor cells is transmitted to the cochlear nuclei via the 8th cranial nerve, and on through the midbrain to the cortex. (Redrawn from Figure 12.3 in [11].) (B) Lateral view of the human brain, with the auditory cortex exposed. The primary auditory cortex contains a topographic map of the cochlear frequency spectrum (shown in kilohertz). (Redrawn from Figure 12.15A in [11].)

Sound is a mechanical vibration whose most defining characteristic is neatly arranged along one dimension—frequency (of waves with alternating high and low pressure). The mechanical structure of our inner ear tidily maps the frequency of sound onto different positions of the basilar membrane in the snail-shaped cochlea [9]. The width and flexibility of this membrane increases with distance from the oval window (the point of entry of sound). The result is that, when the frequency of sound is high, it will produce a peak of vibration near the oval window (Figure 1A); if the frequency is low, the peak of vibration will be nearer the far (apical) end of the cochlea [10]. In this sense, the cochlea processes sound similarly to how a prism acts on white light [11]: it decomposes mixtures of sound frequencies into their components, and maps them onto different spatial positions within the cochlea. The thousands of mechanoreceptors distributed along the basilar membrane need not be tuned to different frequencies, as in color vision, where each receptor type responds most strongly in a particular wavelength range. It is the mechanoreceptors' position in the cochlea that determines which sound frequency will maximally stimulate them [10,12]. This structure is maintained on a higher processing level: fibers in the auditory cranial nerve send the information from the receptor cells to the brain in parallel, i.e., in a first approximation, each receptor cell has its own neuronal “cable” to the cochlear nuclei, and beyond, to the auditory cortex, where we find a complete topographical map of the audible frequency spectrum (Figure 1B), mirroring the mapping of frequencies in the cochlea [11,13,14]. The perception of pitch, then, is arranged along one dimension, as on a piano keyboard. Because of the parallel processing of receptor information from the cochlea, we can hear mixtures of different frequencies, and analyze their components accurately, unless mixtures are very complex. We can identify the tones that a chord is made up of [15]. There are, of course, cases where mixtures have unique properties, e.g., in the case of the intensities of harmonic overtones (integer multiples of the fundamental frequency) that distinguish a C tone produced by a guitar from that of a flute—but we can still identify the fundamental, whose perceived pitch is not altered by the overtones. We would never perceive a mixture of 400 Hz and 800 Hz as an intermediate frequency (e.g., 600 Hz).

## Color Perception in Bees

While these observations are seemingly trivial in the world of sound, the perception of mixtures in color vision is fundamentally different. If we mix yellow with red light, we will see orange—not only will we not be able to tell that the orange light has been produced by mixing two lights, but we will also be unable to distinguish the mixture from monochromatic orange light [16]. Similar mixture phenomena are well established in bees; for example, orange light (with a wavelength of 590 nm) can be mixed with blue light (440 nm) to produce a mixture that is indistinguishable from monochromatic bluegreen light (490 nm) [17,18]. In bees, as in humans, the perception of hues is arranged in a circular fashion around achromatic white or gray [19]. In this circular arrangement, complementary colors (i.e., those opposite on a circle) can be additively mixed to generate a neutral stimulus, e.g., UV light (350 nm) mixed in the appropriate ratio with bluegreen light (490 nm) will be perceived as white by bees [17]. In the auditory system, mixing only two frequencies to produce the perception of white noise is inconceivable! Finally, the circular arrangement of hues also implies that it is possible to mix two ends of the perceptible spectrum to produce a sensation that is not contained in the spectrum: for humans, mixing violet with red light will produce purple, a sensation that has no equivalent in the visual spectrum; a similar unique percept can be produced by facing bees with a mixture of UV and green light [5,17].

We (and other animals) can only ever see one color in a point; additive color mixtures are perceived as intermediates of their generative sources, and it is not possible to identify the physical components of a mixture (e.g., the perception of white can be generated mixing any two complementary colors). In contrast, we can hear several frequencies at once, and identify the components of at least simple stimulus mixtures such as triads [15]. In color vision, but not in hearing, multiple combinations of physical stimuli will generate identical sensations. The reasons for these fundamental differences between the visual and the auditory sensory modalities can be attributed in a straightforward way to the structure of the sense organs as well as post-receptor neural circuits.

**Figure 2:**
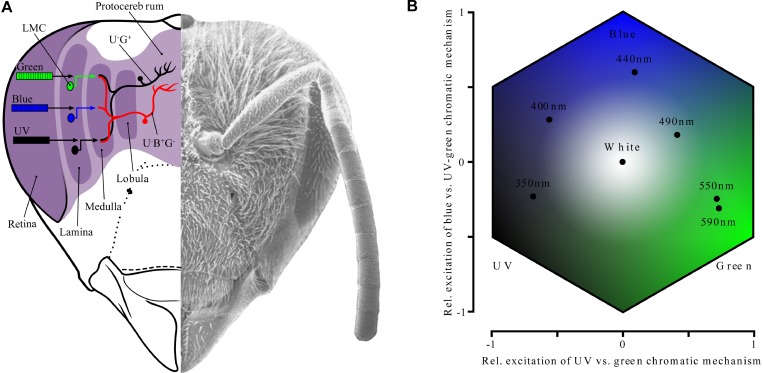
Neuronal Color Coding and Color Space in Bees (A) Frontal view of bee head (scanning electron micrograph) showing essential features of color processing in the brain. Information from the UV, blue, and green receptors is relayed from the first optic ganglion, the lamina, to the second optic ganglion, the medulla, by so-called monopolar cells (LMCs); cell bodies are symbolized by filled circles. These cells feed into color opponent cells (drawn in red and black) found both in the medulla and lobula, either directly or via interneurons. Chromatic opponent cells receive antagonistic input from the different color channels, and project to the protocerebrum. (Image based on [5,18].) (B) Color opponent space for bees, where axes correspond to excitation values of two types of color opponent neurons. Corners correspond to maximum excitation of the UV (lower left), blue (top), and green (lower right) receptors. Color loci of some representative monochromatic lights are shown. Angular position in this space (as measured from the center) corresponds to hue, whereas distance between color loci corresponds to perceived similarity.

In color vision, there aren't thousands of receptor cells each responsible for receiving a narrow range of wavelengths (as there are in the auditory system, with its technique of frequency analysis). Instead, both humans and bees have only three color receptor types, each sensitive to a broad range of wavelengths (Figure 2A). Human color receptors are typically termed blue, green, and red receptors, while those in bees are most sensitive to UV, blue, and green light [5,20]. A single receptor cannot analyze the wavelength of the light it receives: it simply acts as a quantum counter, but the information about the wavelength identity of the quantum is lost on absorption. Any single photoreceptor might respond equally to a medium intensity light in the wavelength range of its peak sensitivity, and to a strong intensity light at the periphery of its sensitive range—hence it cannot disentangle wavelength from intensity [5].

This means that the visual system has to compare the signals from receptors differing in spectral sensitivity. In insects as well as in vertebrates, it does this by means of color opponent neurons (Figure 2A) [18,21,22]. The minimum equipment for an animal with two color receptor types is one type of opponent neuron, receiving antagonistic inputs from the two types of cells. Using such an opponent mechanism, the visual system can “tell” whether there is a stronger signal from the short wavelength receptor or the long wavelength receptor—hence it can extract information about stimulus spectral quality. Theoretically, an animal with n color receptor types needs *n* &minus; 1 chromatic opponent mechanisms [23]. Indeed, behavioral experiments with bees have shown that only two color opponent mechanisms are necessary to explain color discrimination data [18,24]. It is not clear which ones these are—physiologists have found at least seven different types of color opponent neurons in the bee optic lobes [5,18,21], and modeling has shown that almost any combination of two color opponent neuron types is adequate for color coding [24].

Nevertheless, the two-dimensional color opponent space whose axes correspond to excitation patterns of color opponent mechanisms (Figure 2B) has proven extremely useful: distances in such a color space correlate well with behavioral color discrimination data. The color opponent space allows us to predict hue (by assessing angular position) and saturation (by measuring distance from the center), and it can be used to predict the perception of color mixtures, which fall between the loci of the colors used to generate the mixture [5]. Because information about the actual receptor signals is discarded in the very periphery of the sensory system, perception is only based on derived (color opponency) dimensions, which measure differences in receptor signals rather than absolute signals.

## Olfactory Perceptual Space in Bees

Making sense of scents is a considerably messier affair. Odors are hardly presentable on a physical continuum (like the wavelength of light); they are multidimensional entities that can vary from small gaseous molecules to long-chained hydrocarbons [25,26]. Organic compounds vary in carbon chain length and functional group, i.e., the group of atoms that give substances their characteristic properties (e.g., alcohols, aldehydes, ketones, or alkanes). At any moment, the air around an animal may contain hundreds of different airborne substances, which fluctuate with wind, humidity, and multiple other factors [25].

**Figure 3:**
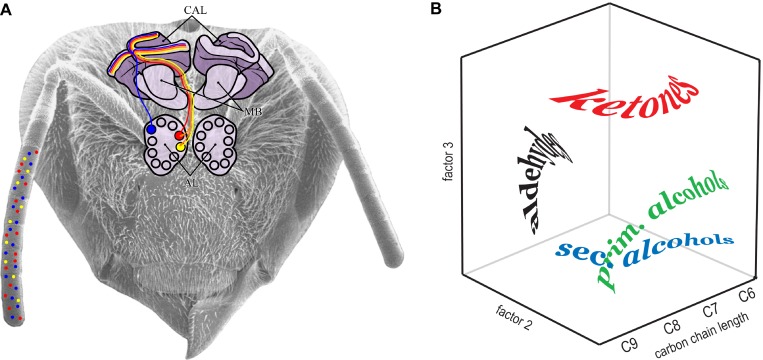
Neuronal Odor Coding and Odor Space in Bees (A) Schematic view of odor processing in the honeybee brain. Some 60,000 odorant receptor cells are distributed along the antenna. These belong to several different types (illustrated with different colors), each responsive to a different set of chemicals. Axons from like receptors project to one or a few glomeruli in the antennal lobe. The glomerular map is organized so that similar odors are mapped to nearby spatial locations (yellow and red), while dissimilar odors stimulate glomeruli located further apart (blue). Axonal projections extend from the antennal lobe to higher processing centers, such as the calyces (CAL) of the mushroom bodies (MB). Some such projections might relay relatively unprocessed sensory information to the mushroom bodies (yellow, red, and blue), while others contain processed information based on lateral interactions between glomeruli (orange, between the yellow and red projections). (B) Putative three-dimensional odor space for bees. Guerrieri et al. [8] trained bees to associate one of 16 odors with a sucrose reward, and then faced bees with the other 15 odors, to see how similarly bees judged these to the training odor. Distances between these substances in a three-dimensional space predict the bee-subjective similarity of the odors. The most important axis corresponds to the carbon chain length of the substances tested; the other two dimensions separate substances according to functional group. Each word illustrates the spatial distribution of a group of substances with like functional group, but varying in chain length. (Image based on Figure 6 in [8].)

On the receptor level, the olfactory system shows a complexity that is unparalleled in any color vision system: the receptor family discovered by 2004's Nobel laureates L. Buck and R. Axel comprises about 1,000 receptor proteins in mammals, each of which only binds a narrow range of airborne molecules [27,28]. Only one of these proteins is expressed per receptor cell, so that there are indeed about 1,000 different odor receptor cell types in the mammalian olfactory epithelium [29]. In insects, the diversity of such receptors is lower, but still impressive: fruit flies appear to have 65 different odorant receptor genes [30]. In honeybees, the number of such genes will be accessible pending the publication of the honeybee genome; so far, screens by H. Robertson (pers. comm.) indicate a number >130. Some 60,000 olfactory receptor cells (of a few dozen different types) are distributed along the honeybee antennae ([31]; Figure 3A). How can the brain extract biologically useful information from such multidimensional sensory input?

The first neuronal center of olfactory information processing, the antennal lobe (or its mammalian analogue, the olfactory bulb) achieves order in two ways. First, axons from like receptor cells (i.e., those that express the same receptor protein and therefore bind the same odorants) project to one or a few glomeruli, i.e., globular, anatomically distinct subunits within the antennal lobe [26]. The number of glomeruli ranges from a few dozen to several hundred, and corresponds roughly to the number of putative olfactory receptor types [25]. The honeybee's antennal lobe contains 160 glomeruli [31]. Individual chemicals reliably activate sets of identified glomeruli [32]. These micro-relays sum up the input from same chemoreceptors, tremendously increasing the signal-to-noise ratio, and thus facilitating reliable odorant detection [26].

A second, and perhaps more remarkable, feature of the antennal lobe is that glomeruli coding for similar substances are located close together, while those that code for distinct scents are spatially segregated [29,32,33,34]. Carbon chain length, for example, is neatly represented in this glomerular map [32]. How the brain achieves such “chemical mapping” is something of a miracle: how would the developing brain “know” which axons belong to receptors that respond to chemically similar substances, so that these can be wired to neighboring glomeruli? In mammals, it was recently found that the receptor proteins that bind odor molecules are also expressed in the axon terminals of the receptor cells [29]. If we assume that similar receptor molecules bind similar odorants, then the developing nervous system could use receptor molecule similarity in the receptor cells' axon terminals to wire up the neural map in the antennal lobe.

But does this neuronal activity map indeed correspond to the olfactory perceptual space? Theoretically, a perceptual space might have as many dimensions as there are distinct receptor types—or it might have as many axes as there are glomeruli with distinct response profiles. Is it possible that olfactory space in bees, then, has several dozen dimensions? To evaluate the structure of olfactory perceptual space, Guerrieri et al. [8] trained honeybees to memorize a wide variety of odors, and then tested how well bees could distinguish these odors from others. They then asked: how many axes must the olfactory perceptual space have, so that distances between odors can be used to predict how similar they will appear to bees? They found that the multidimensional receptor space might be collapsed onto very few perceptual axes: much of the similarity judgments between odors can be explained by a three dimensional space (Figure 3B). The most important axis spreads out scents according to carbon chain length, whereas the other two axes separate the odors according to functional group, i.e., they separate primary and secondary alcohols, aldehydes, and ketones. Distances between odor loci in this three-dimensional space correlate well with the discriminability of the odors to bees. They also correspond to the similarity of activation patterns of the glomerular map [32], although the actual mechanisms that evaluate this similarity remain to be identified. There are a number of complications, however, that indicate that the olfactory perceptual space is unlikely to submit to the relatively simple rules of perception of color and pitch.

Take mixtures of different stimuli, for example. If odor perception followed rules similar to those of color perception, and if it relied exclusively on derived parameters (such as carbon chain length), then mixtures would be perceived as intermediates of their components. A mixture of two molecules differing only in carbon chain length would be perceived as indistinguishable from a single odorant with an intermediate carbon chain length. This remains to be tested, but we conjecture that this is unlikely to be the case. On the other hand, are odor mixtures simply perceived as a compound entity with distinct components (like a triad in vertebrate pitch perception), or are mixtures unique entities that are perceived as fundamentally different from their elements (like “white” in color perception)? Honeybees appear to employ a combination of the two: they can perceive the components of a mixture (and generalize to these components when faced with them individually), but also attach unique properties to the mixture [31,35]. A further complication is that Guerrieri et al. [8] found intriguing asymmetries in the bees' assessments of odor similarities: bees respond as if they find odor A more similar to trained odor B than they find B to trained odor A. It will be difficult to represent these complex phenomena in a simple map.

These phenomena indicate that odor perception cannot be as easily visualized in a low-dimensionality space as other sensory modalities. However, for psychophysicists, just as for motorists navigating novel territory, even a rough guidance map is better than no map, and so this olfactory space is undoubtedly a useful tool to predict the bee-subjective similarity of scents. While the antennal lobe clearly structures the sensory input so as to extract derived chemical properties, the information about the input from individual receptor types might not be discounted in the periphery of the nervous system, as in visual perception, but relayed on to higher processing centers, such as the mushroom bodies and the protocerebrum [36,37]. If both modulated and unmodulated sensory information is available to the neuronal centers that ultimately “decide” on odor similarity, and especially if different individuals attend differently to different kinds of input—e.g., because experience modifies interactions between glomeruli [31]—then the derivation of a static perception space that can be applied to all individuals of a species may be quite challenging.

## Conclusion

Philosophers have correctly pointed out that we cannot actually imagine what it is like to perceive the world through other animals' sense organs [38]. But studies such as those by Guerrieri et al. [8] show elegantly how to attach scales and numbers to the inner worlds proposed by von Uexküll a century ago. Mapping these worlds quantitatively will allow us to compare them between related species operating in different environments, to see how the architecture of perceptual spaces is adapted to mirror biologically useful information from the real world in each species [39]. One of the most exciting future directions is to explore the extent to which such perceptual worlds are not just species-specific, but in fact individual-specific. In the honeybee, learning alters the response patterns of the glomerular map [40]. If the antennal lobe has a dual function in creating the primary dimensions of olfactory perception and storing olfactory memories, then one prediction is that individual experience will alter perception. Similar phenomena have been predicted in other sensory modalities [19,41], but remain to be shown directly by exposing different individuals to different environments during development.
